# *In silico* analysis and expression of a new chimeric antigen as a vaccine candidate against cutaneous leishmaniasis

**DOI:** 10.22038/ijbms.2020.45394.10561

**Published:** 2020-11

**Authors:** Leila Motamedpour, Abdolhossein Dalimi, Majid Pirestani, Fatemeh Ghaffarifar

**Affiliations:** 1 Parasitology Department, Medical Sciences Faculty, Tarbiat Modares University, Tehran, Iran

**Keywords:** Bioinformatics, Leishmania major, Polytope, TLGL, Vaccine

## Abstract

**Objective(s):**

Since leishmaniasis is one of the health problems in many countries, the development of preventive vaccines against it is a top priority. Peptide vaccines may be a new way to fight the Leishmania infection. In this study, a silicon method was used to predict and analyze B and T cells to produce a vaccine against cutaneous leishmaniasis.

**Materials and Methods:**

Immunodominant epitope of *Leishmania* were selected from four TSA, LPG3, GP63, and Lmsti1 antigens and linked together using a flexible linker (SAPGTP). The antigenic and allergenic features, 2D and 3D structures, and physicochemical features of a chimeric protein were predicted. Finally, through bioinformatics methods, the mRNA structure was predicted and was produced chemically and cloned into the pLEXY-neo2 vector.

**Results:**

Results indicated, polytope had no allergenic properties, but its antigenicity was estimated to be 0.92%. The amino acids numbers, molecular weight as well as negative and positive charge residuals were estimated 390, ~41KDa, 41, and 30, respectively. The results showed that the designed polytope has 50 post-translationally modified sites. Also, the secondary structure of the protein is composed of 25.38% alpha-helix, 12.31% extended strand, and 62.31% random coil. The results of SDS-PAGE and Western blotting revealed the recombinant protein with ~ 41 kDa. The results of Ramachandran plot showed that 96%, 2.7%, and 1.3% of amino acid residues were located in the preferred, permitted, and outlier areas, respectively.

**Conclusion:**

It is expected that the TLGL polytope will produce a cellular immune response. Therefore, the polytope could be a good candidate for an anti-leishmanial vaccine.

## Introduction

Leishmaniasis is a parasitic disorder resulting from the obligate intracellular protozoans related to the genus *Leishmania* (1). It has been underestimated worldwide and is more observed in several developing countries and accounts for nearly 12 million infected patients. Cutaneous leishmaniasis (CL) is known as the commonest type of *Leishmania* affecting 0.7 to 1.2 million patients annually (2-4). Such parasites can be transmitted to humans as a result of biting by the infected female sand flies while blood-feeding (5). Leishmaniasis clinical characteristics are associated with several factors, including the species of *Leishmania* as well as the host genetic and immunological features, such as asymptomatic, cutaneous, diffuse cutaneous, mucosal cutaneous, visceral, and post-kala-azar dermal leishmaniasis (6). Leishmaniasis has been shown to be transmitted via various species of *Leishmania* (*Leishmania major* (*L.* *major*)*, Leishmania tropica *(*L. tropica*) and *Leishmania aethiopica* (*L. aethiopica*) in the old world and *Leishmania braziliensis* (*L. braziliensis*) in the new world (7). 

Since cultivation and proliferation of *L. major *in the laboratory are much easier and more practical than *L. tropica*, on the other hand, the vaccine against *L. major *also provides reciprocal immunity against *L. tropica* infection, so the *L. major* species is commonly used for vaccine production. Current management methods are associated with chemotherapy containing pentavalent antimonial, miltefosine, paromomycin, and amphotericin B, which are approved medications for treatment, however, difficulties in the administration of the chemotherapy are a serious problem. Despite awareness of different parasite life cycles, designing a vaccine against leishmaniasis is still under investigation. So far, various antigens from *Leishmania*, such as TSA, LACK, Leif, Gp63, Lmsti1, LPG3, CPA, CPB, KMP, H1, and SMT have been used as vaccine candidates. These antigens have been evaluated so far but they have had a downward effect (8). TSA (Thiol-specific-antioxidant) is a 22.1 KDa protein expressed in promastigotes and amastigotes. It is capable of inducing Th1 immune reaction in BALB/c mice with* L. major *infection. LPG3 (Lipophosphoglycan) is a 95-kDa glycolipid at the cell surface, which is expressed in various promastigote and amastigote forms. It is able to stimulate the production of IgG1 and IgG2a in mice (9)**. ***Gp63* (Glygoprotein63) is a metalloprotease that has an active site on the outer surface of promastigotes and amastigotes stages. This molecule has been identified as an important complement binding site on the surface of the promastigotes. Gp63 actually stimulates IFN-γ production (10). Lmsti1 (stress-inducible protein) is a heat shock protein family found in the amastigotes and promastigotes forms. This protein is able to produce an elevated rate of IFN-γ and low rate of IL4, it enhances potent proliferative responses (11). Recently, bioinformatics and immunoinformatic servers have been developed for identifying appropriate antigens according to their structure, function, and biochemical and biological characteristics. Peptide vaccines may be a new way to fight *Leishmania* infection. A designed multiepitope subunit vaccine can be considered a promising leishmania vaccine candidate. We applied a silico method for prediction and analysis of B cells and T cells to produce a vaccine against CL. So, the efficacy of live recombinant *Leishmania tarentolae *(*L. tarentolae*) in the polypeptide fragment consisting of TSA, LPG3, GP63, and Lmsti1 antigens was evaluated *in silico*.

## Materials and Methods

Using several online servers identifying, as well as assessment of structural, physicochemical, allergenicity, and phosphorylation features were performed.

***Protein sequence retrieval and primary assessment***

The completed amino acid sequence from, *TSA* (GenBank: Accession No.ABX11567.1 ),* LPG3* (GenBank: Accession No. XP_003722150.1), *GP63 *(GenBank: Accession No.ACL01096.2), *Lmsti1* (GenBank: Accession no. XP_001686577.1) *L. major*, were prepared using the National Center for Biotechnology Information (NCBI) protein database, (http://www.ncbi.nlm.nih.gov/protein/) in FASTA format to conduct bioinformatics assessment.

***Immuno-informatics survey about the polytope construct***

*B-cell epitopes forecasting*

Identifying and characterization of B-cell epitopes are essential for designing vaccines, immunodiagnostic evaluations, and antibody synthesis. For identification of continuous B-cell epitopes from the Polytope construct (TLGL), Bcpred online server (http://ailab.ist.psu.edu/bcpred/predict.html) was utilized. The Bcpred technique for epitope predicting employs a subsequence kernel-based SVM classiﬁer (maximum prediction accuracy: 74.57%) (12-14). For Bcpred, epitopes size of 20 amino acids as well as the speciﬁcity threshold of 0.75% were considered. In addition, it identified linear B cell epitopes via physicochemical features. It is able to forecast epitopes at a precision of 58.7%, through the flexibility, hydrophilicity, polarity, and surface features at 2.38 threshold (http://crdd.osdd.net/raghava/bcepred/) (12, 15). Furthermore, using the immune epitope database (IEDB) found at (http://tools.iedb.org/bcell/), the predictions of Bepipred linear epitope (16), hydrophilicity (17), beta-turn (18), surface accessibility (19), flexibility (20), and antigenicity (21) were performed. ABCpred online server (http://crdd.osdd.net/raghava/abcpred/) also was applied. The ABCpred online server can predict B-cell epitope(s) from antigen sequences using an artificial neural network (ANN). ANN can be regarded as the first server designed according to the recurrent neural network (RNN) (a machine-based method) via the fixed size models. The epitopes with an accuracy of 65.93% are predicted through RNN (http://crdd.osdd.net/raghava/abcpred/) (12, 22).

*Cytotoxic T-lymphocyte (CTL) epitope predicting*

For predicting the T-cell epitope, CTLpred online server (23) which is available at http://www.imtech.res.in/raghava/ctlpred/index.html was used. CTLpred is known as a direct technique to predict CTL crucial epitopes and is designed according to the Artificial Neural network (SVM) and support vector machine (ANN) in the subunit vaccine. ANN of 0.51 as well as SVM of 0.36 were regarded as default cutoﬀ values. The best precision obtained by the combined predicting approaches was 75.8% (14)(http://crdd.osdd.net/raghava/ctlpred/about.html).

*T-cell (MHC-I and MHC-II) binding epitopes forecasting*

The IEDB online server (http://tools.iedb.org/mhci/) was used for forecasting MHC-I binding epitopes. For this purpose, H2-Db, H2-Dd, H2-Kb, H2-Kd, H2-Kk, and H2-Ld alleles were selected as the mouse MHC-I molecules. Also, to predict MHC-II epitopes, the (http://tools.immuneepitope.org/mhcii) online server was applied, and H2-IAb, H2-IAd, and H2-IEd alleles as mouse MHC-II molecules were chosen. The half-maximal inhibitory concentration (IC_50_) is determined for every epitope. These epitopes were recognized through online software for strong binding with the MHC-I and II molecules. In addition, the IEDB-recommended technique using 15 amino-acid-long peptides and the percentile rank specific yield were utilized for forecasting. 

***Fusion peptide to produce the final polytope construct***

Using the IEDB online server, B and T-cell epitopes with high affinity and different segment arrangements for each gene were selected, then linked together using a flexible linker and the final TLGL was designed. 

***Immuno-informatics assessment of the polytope construct***

*Physico-chemical parameters evaluation*

TLGL common characteristics, such as molecular weight (MW), instability index (II), *in vitro* and *in vivo* half-lives, theoretical isoelectric point (PI), amino acid constitution, positive and negative residues final value, extinction coefficient, II, aliphatic index, and grand average of hydropathicity (GRAVY) were investigated through the protparam tool (https://web.expasy.org/protparam/) (24).

*Phosphorylation and acylation positions of Polytope construct*

Phosphorylation was assessed by NetPhos 3.1 (http://www.cbc.dtu.dk/services/Netphos/) and acylation positions of TLGL by CSS-Palm server (http://csspalm.biocuckoo.org/online.php)(25). 

*Secondary and tertiary (3D) structures analysis*

Through the Garnier-Osguthorpe-Robson (GOR) 2D structure forecasting technique, secondary structures of polyepitope construct (TLGL) were predicted (https://npsa-prabi.ibcp.fr/cgi-bin/npsa_automat.pl?page=/NPSA/npsa_gor4.html) (26). 

SCRATCH (http://scratch.proteomics.ics.uci.edu/) predicted the residues relative solvent accessibility (27). However, DiNNNA program was applied to identify disulfide bonds (http://clavius.bc.edu/~clotelab/DiANNA/) (27, 28). SWISS-MODEL was employed for prediction of the 3D structure of TLGL (https://swissmodel.expasy.org/) (25, 29).

*Tertiary modeled structure reﬁnement and validation*

A two-step refinement procedure was conducted. Initially, the most appropriate 3D pattern was obtained from SWISS-MODEL and refinement of TLGL was done by ModRefiner (30) (https://zhanglab.ccmb.med.umich.edu/ModRefiner/). For ﬁnding possible errors in the primary 3D models, Ramachandran plot analysis (http://mordred.bioc.cam.ac.uk/rapper/rampage.php) was utilized (31).

*Antigenicity and allergenicity prediction*

Using VaxiJen v2.0 and ANTIGENpro predicting the antigenicity of TLGL was done (32).

VaxiJen 2.0 (http://www.ddg-pharmfac.net/vaxijen/VaxiJen/VaxiJen.html) (33) as a novel alignment-free method to predict antigen, works in accordance with the auto-cross-covariance (ACC) change of peptide sequences to similar vectors from the main amino acid features. Its precision (http://www.ddg-pharmfac.net/vaxijen/VaxiJen/VaxiJen_help.html) varies between 70% and 89% based on the target organism. ANTIGENpro is known as a sequence-oriented, alignment-free, and pathogen-independent prediction model for protein antigenic potential at http://scratch.proteomics.ics.uci.edu/ used for generating an antigenicity index. It was firstly used to predict the total protein antigenicity trained through reactivity values from protein microarray assessment (http://scratch.proteomics.ics.uci.edu/explanation.html#ANTIGENpro). For predicting the TLGL allergenicity, the AlgPred web server (http://www.imtech.res.in/raghava/algpred/) was applied, in which allergens are predicted by the resemblance of the identified epitope with protein’s regions. It also predicts according to the six approaches. In this study, the hybrid method (SVMc+IgE epitope+ARPs BLAST+MAST) at a precision of 85% and -0.4 threshold was employed (34).

*Predicting protein solubility*

Limited data is available regarding solubility in recombinant proteins. SOLpro server was employed for prediction solubility of heterologous protein following overexpression (http://scratch.proteomics.ics.uci.edu/)(35).

***Gene expression in L. tarentolae***

*Cloning of TLGL in the expression vector*

The chimeric sequence of TLGL was synthesized by the Mede Bioeconomy Co. (Iran) into the pEGFP-N1 plasmid at the enzyme site of SalI, BglII, and NotI. First, the chimeric sequence was transmitted to *E. coli *TOP10 strain. The plasmids pEGFP-N1 and pLEXY-neo2 were digested by BglII and SalI restriction enzymes and NotI and BglII for secretory and cytosolic expression, respectively. In the next step, the enzymatic digestion products were ligated in pLEXY-neo2 by T4 Ligase enzyme. 

*Transfection of pLEXY-TLGL into L. tarentolae*

The *L. tarentolae *Tar II (ATCC 30143) strain was grown in RPMI-1640 medium (Gibco) treated with 10% fetal calf serum (FCS, Gibco) affected by heat inactivation (pH: 7.2 and 26 ^º^C). Washing of the 3.5×10^7^ log-phase parasites was done to transfect followed by resuspending in 350 μl of electroporation buffer (pH 7.5), including Hepes (21 mM), NaCl (137 mM), KCl (5 mM), Na_2_HPO_4_ (0.7 mM), and Glu (6 mM). It was fused with 50 µg of H_2_O containing linearized pLEXSY-TLGL (10 μg) with *SwaI* restriction enzymes (Fermentas, USA), stored in ice within 10 min followed by electroporation using Bio-Rad Gene Pulser Ecell (450 V and 500 µF). Afterward, the electroporated promastigotes were added to 5 ml of RPMI-20% FCS medium without any selective antibiotics and incubated for 24 hr at 26 ^º^C. Cell growth, which was highly resistant against geneticin (G418) (Sigma, USA) was seen 7-10 days later (36).

***RNA extraction and reverse-transcription PCR (RT-PCR)***

Total RNA specimens from recombinant *L. tarentolae* promastigote types with the Sina Clon RNX Plus Kit (cat. No. RN7713C) as instructed by the producer. The RNA concentration and its quality were measured via UV absorbance (Thermo Scientific NanoDrop 2000) and electrophoresis on 2% agarose gel. The cDNA was synthesized by using the ROJE kit (cat. no. EB983028-S) and was amplified by specific forward (CCGACTGCAACAAGGTGTAG) and reverse (CATCTATAGAGAAGTACACGTAAAAG) primers. According to the manufacture’s protocol, in the first step, 5 μl of template (total RNA: control or recombinant) was mixed with 1 μl Oligo dt primer (100 pmol) and 6 μl DEPC-treated water, then, the mixture was kept at 65 ^°^C for 5 min, then put on ice for 2 min. In the second step, 4 μl reaction buffer (5X), 1 μl ribonuclease inhibitor (50 units/ µl), 2 μl 10 mM dNTP (1 mM final concentration), and 1 μl reverse transcriptase were mixed and the final volume of two steps (20 μl ) was kept at 42 °C for 30-60 min and finally at 85 °C for 15 Sec. Finally, the RT-PCR product was investigated by electrophoresis on 1.2% agarose gel (37).

***Prediction of mRNA structure***

The mfold tool (http://unafold.rna.albany.edu/?q=mfold) was employed for prediction of RNA secondary structure to define the free energy accompanied by the 5′ end in the mRNA of the chimeric gene.

***Western blot analysis***

Promastigote forms of transfected recombinant *L. tarentolae* and the wild type parasite were removed with centrifugation (3000 rpm/15 min) followed by washing in PBS. The cell pellets lysis was done in 5x SDS-PAGE sample buffer (4.5 mM Tris–HCl, pH 6.8, 10% glycerol, 2% SDS, 5% 2-mercaptoethanol, 0.05% bromophenol blue) on ice for 30 min and followed by boiling within 5 min. SDS-PAGE 12.5% was used for running the specimens. Afterward, transitioned to 0.2 μm Immune-Blot™ polyvinylidene difluoride membranes (Cat No: 162-017777; Bio-Rad Laboratories, CA, USA) (38) and Western blotting was used according to the standard procedure (39).

## Results

***B and T-cell epitopes prediction for synthesis of polytope construct***

**Figure 1:**
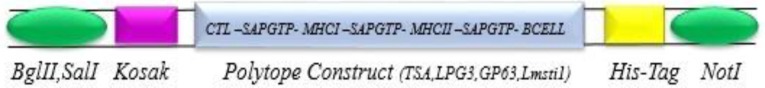
Schematic scheme of a polytope construct with restriction sites

In this study, for each gene, one epitope of T-cell, B-cell, and MHCI, and two epitopes of MHCII were identified using the IEDB online service with a high score to be expressed as recombinant proteins by using the standard molecular biology method, were selected. After selecting twenty favorable epitopes, they were connected with a SAPGTP linker fragment and the final TLGL with a molecular weight of ~41 kDa was obtained (Figure 1). The characteristics are shown in Table 1.

***General basis of polytope constructs predictions***

**Figure 2:**
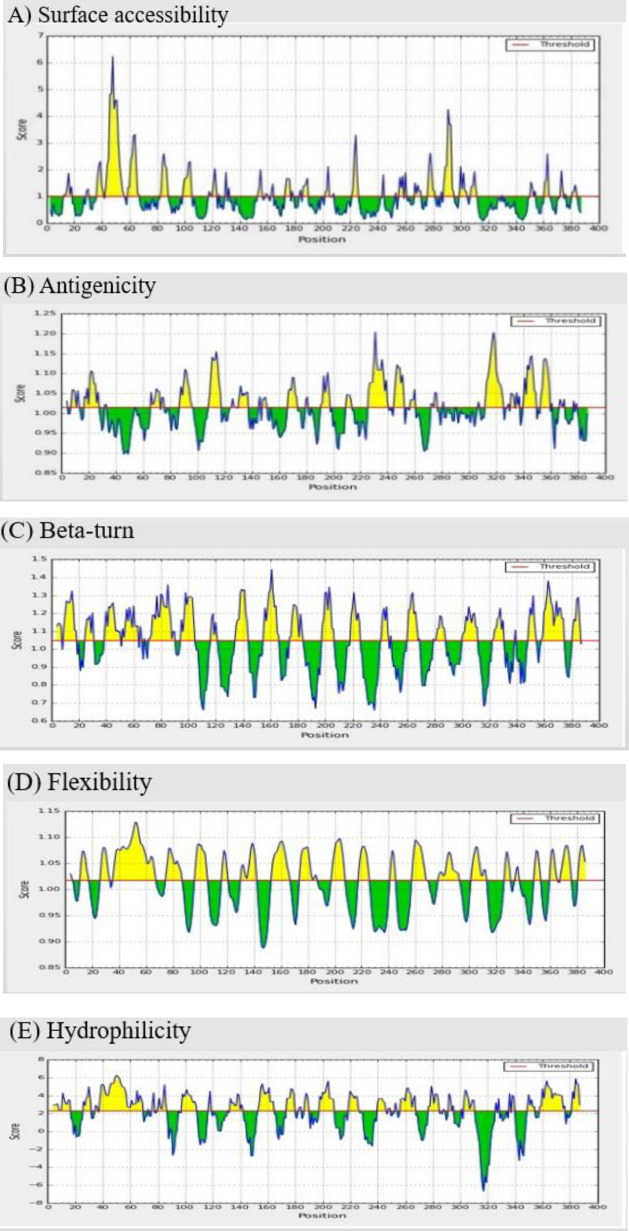
Tendency scale plot from polytope construct (TLGL). A) Surface accessibility; (B) Antigenicity; (C) Beta-turn; (D) Flexibility; (E) Hydrophilicity. The threshold/mean rank is shown by a horizontal red line. Desirable areas of interest properties are shown in yellow (over the threshold). Green (below the threshold) demonstrates undesirable areas of interest features

Table 2 shows the results of prediction for continuous B-cell epitopes by the Bcepred online server. Such potential epitopes have been shown to be crucial for TLGL antigenic properties. The antigenicity, surface accessibility, flexibility, beta-turn, and hydrophilicity average score (threshold) on TLGL with IEDB were determined as 1.015, 1, 1.018, 1.047, 2.265, respectively (Figure 2).

***Secondary and tertiary structure analysis***

**Figure 3:**
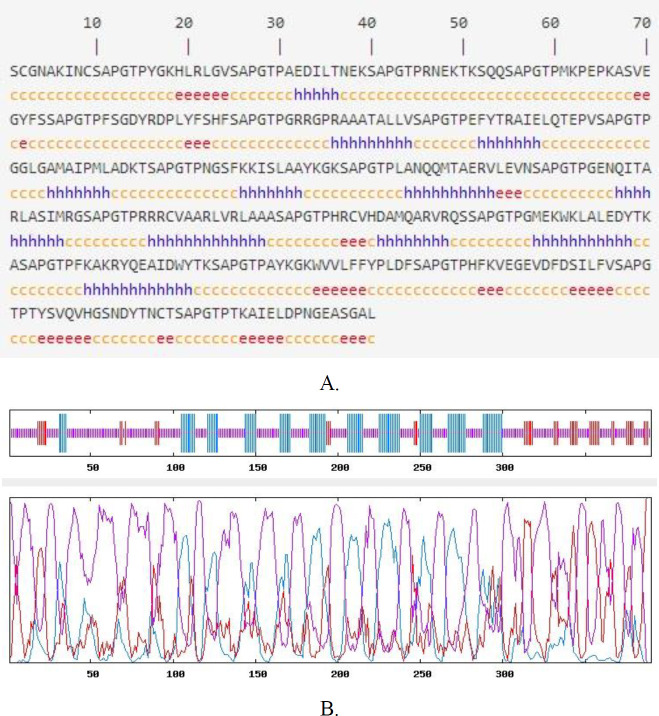
(A) Prediction of the secondary structure of TLGL with GOR4 online service (https://npsa-prabi.ibcp.fr/cgi-bin/npsa_automat.pl?page=npsa_gor4.html). H, helix, e, extended strand, and c, coil; (B) The graph indicating TLGL secondary structure predicting with GOR 4

**Figure 4:**
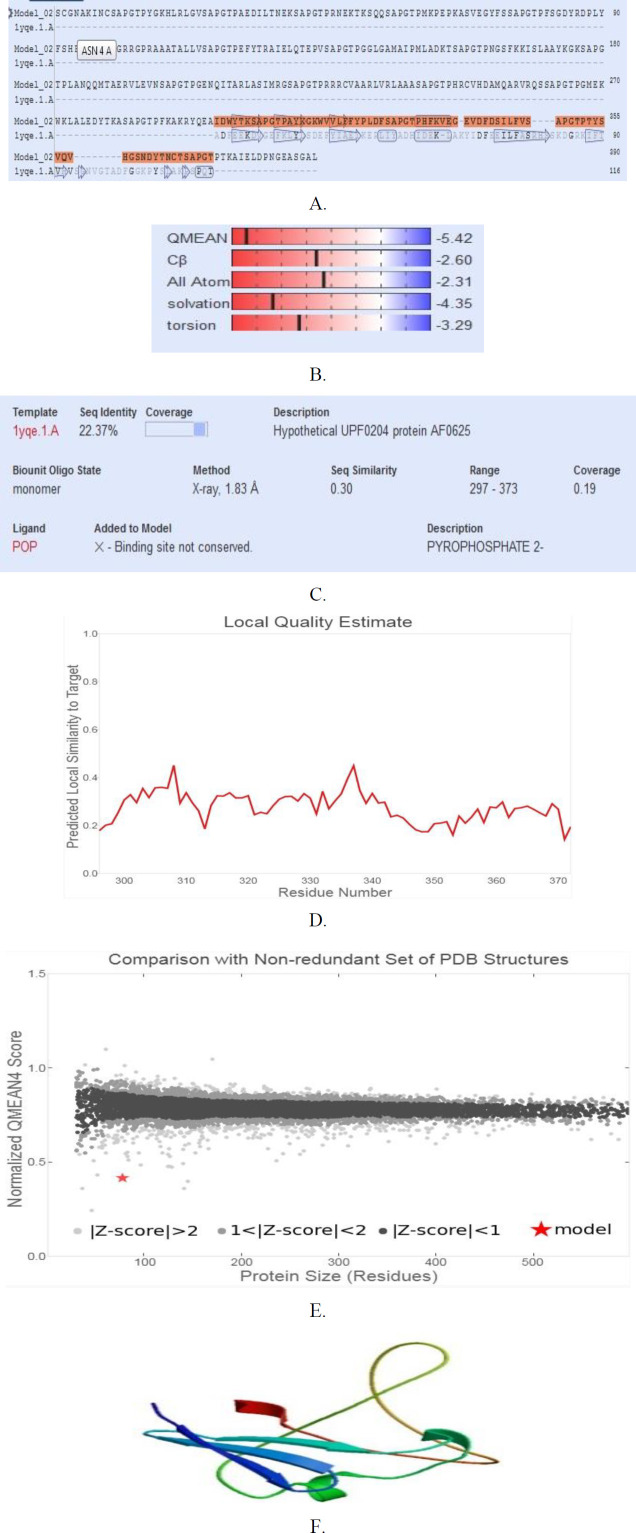
The SWISS-MODEL server product (https://swissmodel.expasy.org/). (A) SWISS-model template alignment; (B) global model quality estimation; (C) Sequence similarity as well as coverage; (D) local model quality estimation; (E) Comparing with non-waste PDB structure; (F) produced a 3D model for polytope construct (TLGL)

According to the GOR4 online service, it was found that the secondary structure consists of 390 amino acids, 25.38% (99/390) alpha helix (H), 12.31% (48/390) extended strand, and 62.31% (243/390) random coil in TLGL. Secondary structure prediction was represented in Figure 3. SCRATCH server was used to estimate the distribution of the solvent accessibility area by the residual models’ hydrophobicity as well as polar characteristics. Based on the findings, solvent availability was acceptable, and the remainder is available in every TLGL domain. To predict the functional characteristics and 3D structure of the polytope construct, the online server of SWISS-MODEL was utilized. After predicting, forming 2D models was done regarding our sequence, and pattern including the maximum sequence identification was selected. It had a 22.37% sequence identity indicating the maximum coverage of the proposed patterns by SWISS-MODEL. The output of SWISS-MODEL is indicated in (Figure 4). In the present study, the DiNNNA online server was used to identify disulfide bonds. The results showed that there are 5 cysteine’s in TLGL. Our sequence cysteines are used to form disulfide bonds at positions 2 - 9, 9 – 228. More details can be found in Table 3. 

***Refining the 3D model structure and verification***

**Figure 5.**
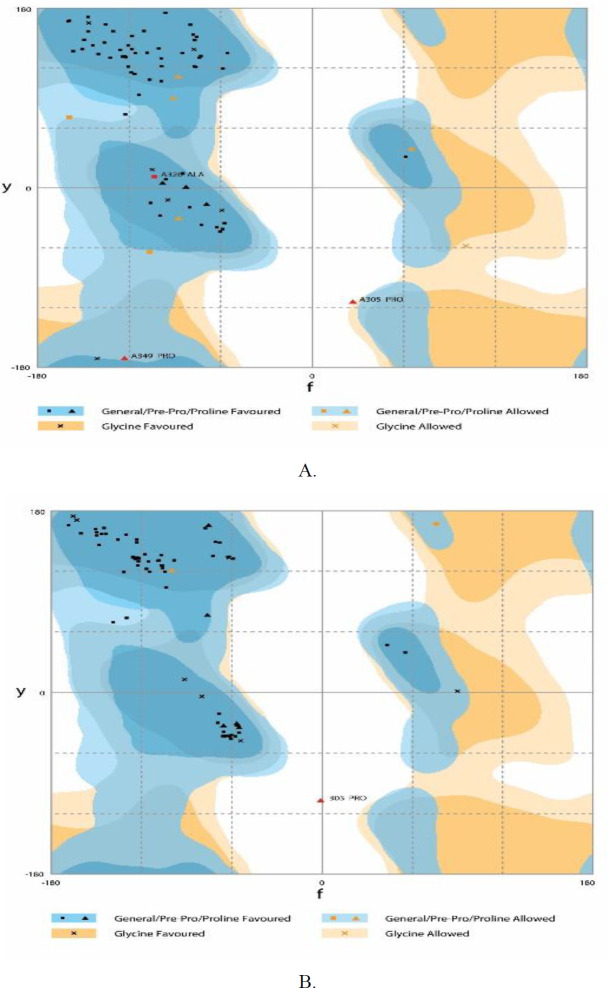
Validating the polytope construct tertiary structure by Ramachandran plot. Ramachandran plot analysis, the overall preferred and Pre – Pro preferred areas are in Dark blue. Pale blue color demonstrates the overall allowed as well as Pre – Pro allowed areas. Glycine favored and allowed areas are represented by dark and pale orange, respectively. White color illustrates the disallowed areas. (A) Analysis of statistics Ramachandran plot for the primary model. (B) The RAMPAGE outcome after refinement

Validation of the reﬁned TLGL 3D structure, before and after refinement was done through generating the Ramachandran plot via RAMPAGE. Figure 4 shows Ramachandran plot productions before as well as following refining. Before refining polyepitop (TLGL), Ramachandran plot indicted 86%, 9.3%, and 4% residues in the preferred, permitted, and outlier areas, respectively. Following refining the pattern, 96% residues were found in the preferred area, while 2.7% of them were in the allowed and 1.3% were found in the outlier areas (Figure 5). 

***Physicochemical parameters evaluation***

ExPASy ProtParam was applied to predict different physiochemical features related to the ultimate polytope product. This construct contains 390 amino acid residues (molecular weight: ~ 41 KDa and theoretical pI: 9.36). The final rate of negatively (Asp+Glu) and positively (Arg+Lys) charged residues was 30 and 41, respectively. Extinction coefficients using M^-1^ cm^-1^, at 280 nm determined in water obtained 36120 (36120 M^-1^ cm^-1^). The half-life estimated 1.9 h (mammalian reticulocytes, *in vitro*) over 20 h (yeast, *in vivo*), and over 10 h (*E. coli*, *in vivo*). II was estimated at 28.70, which indicates the constant protein. The aliphatic index was 57.72 and its higher values are representative of a constant protein in varied temperature conditions. In addition, the GRAVY score of -0.444 was obtained for vaccine structure. The negative GRAVY scores are indicative of the hydrophilic protein as well as favorable interaction with the adjacent H_2_O molecules.

***Phosphorylation and acylation positions of the polytope construct***

**Figure 6:**
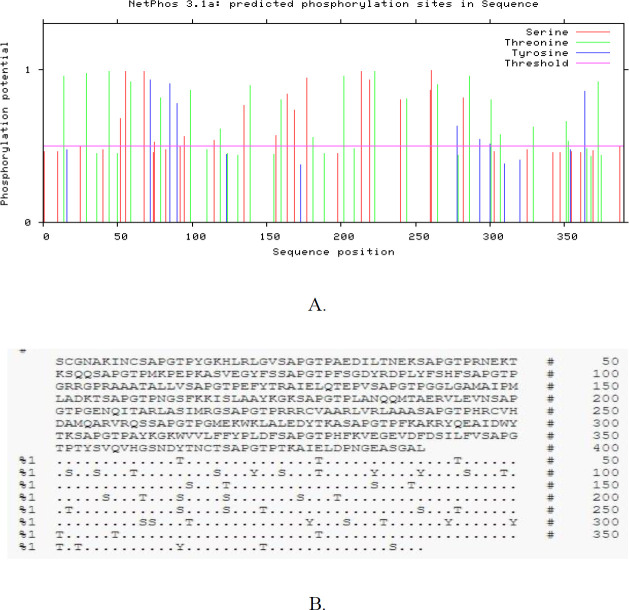
Bioinformatics assessment related to the phosphorylation and acylation areas of polytope construct (TLGL). (A) Prediction of phosphorylation sites in protein construct; (B) If the remnant is not phosphorylated, either due to lower score than the threshold, or owing to no Ser, Thr, or Tyr remnant, such area is denoted using (‘.’). The remnants characterized by predicting scores more than the threshold indicated as ‘S’, ‘T’ or ‘Y’, respectively

In the current study, to survey the phosphorylation and acylation sites of TLGL, NetPhos 3.1, as well as CSS-Palm servers, were employed, respectively. It is significant that there were 45 phosphorylation areas (Ser:17, Thr: 21, Tyr: 7) (Figure 6) and 5 acylation sites (Table 4) in our TLGL. Therefore, in our sequence, there are 50 potential Protein Post-Translational Modification (PTM) sites.

***Antigenicity, allergenicity and solubility prediction***

TLGL antigenicity was estimated at 0.922 using Vaxijen at 0.5% threshold for parasite pattern as well as 0.943% using ANTIGENpro. Algpred server was applied for forecasting the protein allergenicity, indicative of the non-allergenic TLGL. Then, the propensity of the heterologous peptide solubility by using the SOLpro server was estimated at 0.932807.

***Predicting mRNA construct***

**Figure 7:**
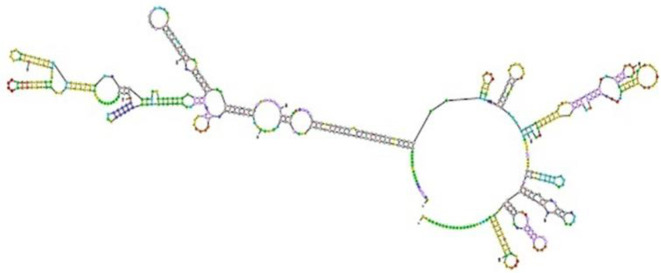
The predicted mRNA construct without hairpin and pseudoknot at the 5′ end

Using mfold the least release energy required to form mRNA secondary structures was estimated. The ΔG of the forecasted TLGL was *-164.50* kcal/mol. No constant hairpin/pseudoknot was formed by the initial nucleotides at the 5′ site (Figure 7).

***Generating recombinant L. tarentolae expressing TLGL***

**Figure 8:**
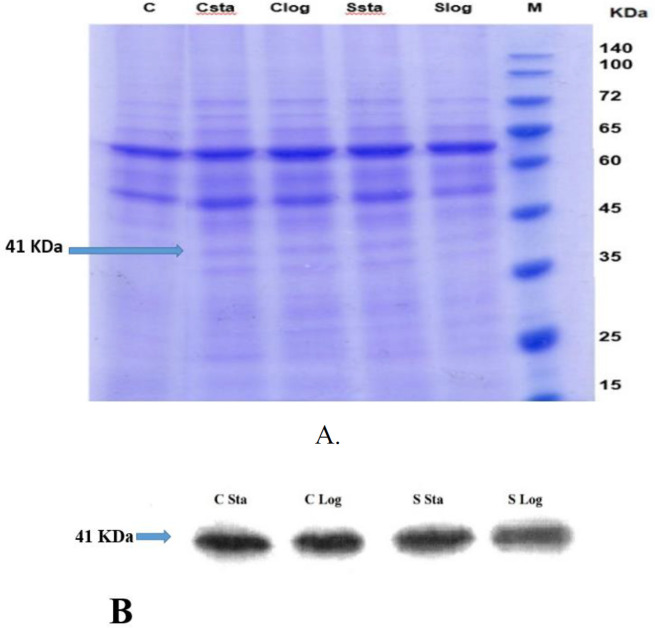
SDS-PAGE analysis of the level of expression of chimeric sequence that successfully subcloned to pLEXY-neo2 and expressed in *Lishmania tarentolae*. Lane 1, Protein molecular weight marker (10–140 kDa); Lanes 2: Logarithmic phase secretory sample, Lanes 3: Stationary phase secretory sample, Lanes 4: Logarithmic phase cytosolic sample, Lanes 5: Stationary phase cytosolic sample and Lanes 5: Control (*L. tarentolae* secretory sample) (A). Western blot analysis ( B)

The chimeric sequence was successfully subcloned to pLEXY-neo2 and expressed in *L. tarentolae *after electroporation. The cDNA of recombinant promastigote of *L. tarentolae *was amplified using a specific primer. It showed that the product is 1170 bp. The results of SDS-PAGE and Western blotting revealed that cytosolic and secretory samples express the recombinant protein with ~ 41 kDa of molecular weight (Figure 8). 

**Table 1:** The epitopes selected from different antigens of Leishmania according to B-cell, MHCI and MHCII alleles

Gene	Epitope B-cell ^a^	MHCI^b^	MHCII^c^	CTL^d^
TSA	AYKGKWVVLFFYPLDF	MKPEPKASVEGYFS	GGLGAMAIPMLADKT NGSFKKISLAAYKGK	SCGNAKINC
LPG3	HFKVEGEVDFDSILFV	FSGDYRDPLYFSHF	LANQQMTAERVLEVN GENQITARLASIMRG	YGKHLRLGV
GP63	TYSVQVHGSNDYTNCT	GRRGPRAAATALLV	RRRCVAARLVRLAAA HRCVHDAMQARVRQS	AEDILTNEK
LMSTI	TKAIELDPNGEASGAL	EFYTRAIELQTEPV	GMEKWKLALEDYTKA FKAKRYQEAIDWYTK	RNEKTKSQQ

^a^ BCPREDS as well as ABCpred servers were employed for forecasting linear B-cell binding epitopes. Predicted B-cell epitopes are scored according to the values achieved from repeatedly trained neural networks and the more peptide score represents the more possibility of an epitope

^b^ Using the IEDB online software the elevated ranked as well as distributed binding epitopes to MHC-I alleles

^c^ Using the IEDB online software the elevated ranked as well as distributed binding epitopes to MHC-II alleles

^d^ Using CTLpred servers, high-ranking epitopes were identified

**Table 2:** The predicted epitope sequences in protein construct (TLGL) by several predicting factors using Bcepred server

Predicting factor	Epitope sequences
Hydrophilicity	PGTPAED; TNEKSAPGTPRNEKTKSQQSAPGT; KPEPKAS; SGDYRDP; GTPGRRG; SAPGTPE; ADKTSAPGTPNGS; KGKSAPGT; PGTPGENQ; RGSAPGT; RQSSAPGT; EDYTKAS; TKSAPGT; QVHGSNDYTNCTS; DPNGEASGA
Flexibility	DILTNEKSAPGTPRNEKTKSQQ; FSAPGTPGRRGP; SAPGTPNGSF; AAYKGKSA; SAPGTPG; SIMRGSAPGTPRR; MQARVRQSS; LDPNGEA
Accessibility	PGTPYGKHLR; PGTPAEDILTNEKSAPGTPRNEKTKSQQSAPGTPMKPEPKASVE; PFSGDYRDPLYF; PGTPGRRGPRAA; TPEFYTRA; ADKTSAPGTPNGSFKK; AAYKGKSAPGTPLANQQMTAER; PGTPGENQITAR; APGTPRRRCV; APGTPHR; MQARVRQSSAPG; PGMEKWK; ALEDYTKASAPGTPFKAKRYQEAIDWYTKSAPGTPAYKGKW; PGTPTYS; HGSNDYTNCT; APGTPTKAIELDPNGEAS
Turns	VHGSNDYTN
Exposed Surface	GTPRNEKTKSQQS; PMKPEPKAS; TPFKAKRYQE
Polarity	PYGKHLRLGV; GTPRNEKTKSQQ; PMKPEPKASVE; PGRRGPRAA; TAERVLEVN; PGTPRRRCVAAR; TPHRCVHDA; PGMEKWKLA; TPFKAKRYQEAI; TPHFKVEGEVDFD
Antigenic Propensity	KHLRLGVS; PLYFSHFS; GTPHRCVHD; KGKWVVLFFYPLDF; VDFDSILFVS; PTYSVQVHGS

**Table 3:** Disulfide bond predictions for different cysteines sequences positioning using DiNNNA online server

Disulfide bond scores			
Cys sequence positioning	**Length **	**Bond**	**Value **
2 – 9	7	XXXXSCGNAKI-NAKINCSAPGT	0.01105
2 – 228	226	XXXXSCGNAKI-TPRRRCVAARL	0.01111
2 – 248	246	XXXXSCGNAKI-GTPHRCVHDAM	0.01129
2 – 367	365	XXXXSCGNAKI-NDYTNCTSAPG	0.01453
9 – 228	219	NAKINCSAPGT-TPRRRCVAARL	0.98394
9 – 248	239	NAKINCSAPGT-GTPHRCVHDAM	0.99964
9 – 367	358	NAKINCSAPGT-NDYTNCTSAPG	0.99976
228 – 248	20	TPRRRCVAARL-GTPHRCVHDAM	0.01277
228 – 367	139	TPRRRCVAARL-NDYTNCTSAPG	0.06127
248 – 367	119	GTPHRCVHDAM-NDYTNCTSAPG	0.67992
Weighted matching			
Predicted bonds			
9 – 228		NAKINCSAPGT – TPRRRCVAARL	
248 – 367		GTPHRCVHDAM – NDYTNCTSAPG	

**Table 4:** The protein construct acylation areas

Code	Area	Peptide	Value
Unnamed	2	******SCGNAKINC	21.429
Unnamed	9	CGNAKINCSAPGTPY	14.067
Unnamed	228	PGTPRRRCVAARLVR	1.436
Unnamed	248	APGTPHRCVHDAMQA	5.533
Unnamed	367	GSNDYTNCTSAPGTP	3.757

## Discussion

Bioinformatic tools decrease the time and cost of diagnosis which is needed to appropriate B and T cell immune epitopes while increasing the research accuracy (40). Recently, for various vaccine designs, immuno-informatics and bioinformatic tools, in various fields have been successfully employed (41-49). Adu-Bobie *et al.* (2003) and Delany *et al.* (2013) designed a reverse vaccinology strategy that was effective against the serogroup B *Neisseria meningitides* (50, 51). Also, *Meningococcus* B was the first pathogen identified by reverse vaccinology. A wide range of vaccines were made based on immunoinformatics and revers vaccinology like effective vaccines against *Streptococcus pneumoniae, Chlamydia pneumonia, Staphylococcus aureus *and many others (50). Multi-epitope leads to showing a repetitive antigen on the surface of the vaccine and increases the immune response compared to single immunogens (43). For *the Leishmania* vaccine, a group designed a DNA vaccine-based *L. major* polytope using GP63, LACK, CPC antigens successfully (52). To produce peptide-based vaccines, researchers conducted a study to identify T cell (*MHCII)* epitopes using antigens such as LPG3 and NH and to produce a vaccine against *Leishmania donovani* (53). Vakili *et al.* (2018) designed a potent multiepitope peptide for a vaccine against *Leishmania infantum *(*L. infantum*). They used histone H1, sterol 24-c-methyltransferase (SMT), leishmania-specific hypothetical protein (LiHy), and leishmania-specific antigenic protein (LSAP) antigens for this vaccine (54). In the present study, four *L. major *antigens were used to design a polytope construct. The Expasy ProtParam server was used for assessing the TLGL physicochemical features (https://web.expasy.org/protparam/) (24). It contained 575 amino acids with a molecular weight of ~41 kDa, which showed good antigenicity. As mentioned before, antigens of less than 5-10 kD were regarded as poor immunogens (55). The construct aliphatic index was 57.72 and its GRAVY was -0.444. The elevated aliphatic index is a presentation of high thermostability of protein that was determined by the final relative size of aliphatic side chains (56). Also, the negative GRAVY value of the polytopconstruct demonstrates the hydrophilic feature of the protein resulting in more favorable contact with adjacent H2O molecules (57). PTM is essential in cellular management strategies (58). Therefore, for analyzing the acylation areas, CSS-Palm was used. Also, we used NetPhos 2.0 for analysis of the phosphorylation areas of TLGL**. **The results show that TLGL contains 45 phosphorylation areas (Ser: 17, Thr: 21, Tyr: 7), as well as five acylation areas, in which 50 potential PTM areas can be seen in TLGL. These sites may regulate the function of several proteins and may affect their activities. The secondary structure of TLGL was studied with the GOR IV method. The results showed that the analysis of protein secondary structure is remarkably effective for epitopes (59). The alpha-helix and beta-turn located inside the protein include elevated hydrogen-bond energy that makes strong interactions with antibodies and therefore protects the protein structure (60). Our study showed that this protein contains 25.38% alpha-helix, 12.31% extended strand, and 62.31% random coil. One of the important points is to discover the relationship between the four antigens and their function to determine the structure of the chimeric proteins, because structural information is important for predicting immunogenicity. In this study via SWISS-MODEL, the 3D structure of protein sequence revealed a suitable spatial structure and was then refined using ModRefiner. Ramachandran plot is essential to evaluate the experimental structure’s quality as well as predict the protein’s biological role (61, 62). Based on the results, the 3D TLGL quality has increased significantly, indicating an improvement in the product structure quality compared with the original one (54). Restriction of antigen design reduces protein expression in the host (63). Furthermore, the mRNA stability commonly is associated with MFE, indicating the consistency of a complete or near-complete secondary stem-loop hairpin structure of pre-miRNAs. It is significant that the RNA molecule characterized by low MFE is constant (64). Sequences of TLGL precursor mRNAs demonstrate negative MFEs of -164.50 kcal/mol. Protein antigenicity makes that protein detectable via the immune system. The antigenicity of polytope constructs with Vaxijen and ANTIGENpro was estimated at 0.922 and 0.943%, respectively. These results showed that TLGL is likely to have antigenic features and is capable of adequately stimulating the T and B cell immune response. Also, Algpred server showed that the designed polytope is not allergenic. In this study for expression of the designed polytope, *L. tarentolae *was used. This parasite was not pathogenic for mammals. Special features of this organism such as high growth rate, inexpensive growth conditions, non-pathogenicity, appropriate glycosylation, and ultimately the successful expression of several different proteins, made this parasite a potential host for the expression of heterologous glycoproteins and were anticipated as substitutes for mammalian cells in recombinant protein expression processes (65-68). To predict a prominent vaccine candidate to prevent leishmaniasis, the combined bioinformatics, online servers, and various software were used for predicting possible B and T cells. The forecasted sequences immunogenicity should be approved using various bioinformatics methods in an appropriate mouse model, therefore, further investigations using *in silico* and *in vivo* patterns must be done in the future for estimating the potency of the polytope as an eventual vaccine option (7, 69).

## Conclusion

We have successfully presented a protein construct retrieved from the TSA, LPG3, GP63, and Lmsti1 antigens of *L. major *in *L. tarentolae*. It is expected that the TLGL polytope will produce a humoral and cellular immune response in the animal model. Of course, the immunization property of polytope designed with different informatics approaches could be evaluated in a suitable mouse model.
